# A postmortem study on indigestible foreign bodies in the rumen and reticulum of ruminants, eastern Ethiopia

**DOI:** 10.4102/ojvr.v82i1.881

**Published:** 2015-05-29

**Authors:** Seifu Negash, Berhanu Sibhat, Desie Sheferaw

**Affiliations:** 1 Hawassa University School of Veterinary Medicine, Hawassa, Ethiopia; 2 Haramaya University College of Veterinary Medicine, Haramaya, Ethiopia

## Abstract

A cross-sectional study was conducted on ruminants (cattle, sheep and goats) slaughtered at Haramaya University and Haramaya municipal abattoirs from November 2013 to April 2014 in Haramaya, eastern Ethiopia. The objective of the study was to identify types and estimate the prevalence of foreign bodies in the rumen and reticulum of domestic ruminants in the area. From 810 randomly selected study animals, 422 (52.1%) were found to have foreign bodies. Of the 332 cattle, 193 sheep and 285 goats examined, 144 (43.4%), 109 (56.5%) and 169 (59.3%) respectively were found with various types of foreign bodies. The prevalence of foreign bodies was significantly (χ^2^ = 17.53, *p* < 0.05) higher in sheep (59.3%) and goats (56.7%) than in cattle (43.4%). Overall the prevalence of foreign bodies in study animals with poor body condition was significantly higher (χ^2^ = 38.57, *p* < 0.05) than in those with medium and good body condition. A higher percentage of foreign bodies occurred in the rumen alone (87.9%) than in the reticulum alone (5.0%), with the rest present in both. Significantly higher proportions of foreign bodies were observed in the rumen of cattle (χ^2^ = 332, *p* < 0.05), sheep (χ^2^ = 193, *p* < 0.05) and goats (χ^2^ = 285.0, *p* = 0.000) than in the reticulum. Plastic was the most commonly encountered (79.2%) foreign body, followed by cloth (15.3%) and rope (12.3%). In addition, metal (0.9%) and calcified material and/or stone (1.0%) were found in the reticulum of cattle. Lack of a plastic waste disposal system in the area as well as communal/free grazing of livestock in highly waste-polluted areas seemed to be major factors in the high occurrence of foreign bodies in ruminants. To change this, collaborative intervention schemes involving professionals, policy makers, livestock keepers and environmental activists are needed.

## Introduction

Livestock are a source of high-quality protein (meat, milk and eggs) and also contribute to the economic welfare of people by providing hides, skins, fertiliser, and power and traction for agricultural purposes, increasing the productivity of smallholdings (Torr *et al.* 2003). They are also a ‘living savings bank’, serving as a financial reserve for periods of economic distress and crop failure and as a primary source of cash income (International Livestock Research Institute 1999). However, indigenous livestock in Ethiopia have not been fully exploited due to their lower productivity, the main reasons for this including poor nutrition, disease and poor genetic make-up. Ingestion of indigestible foreign bodies is mainly associated with nutritional deficiencies, environmental pollution and poor feeding management, and causes various problems in the rumen and reticulum of ruminants (Jones, Hunt & King 1997). Harmful effects include reduced feed intake, failure to absorb volatile fatty acids, reduced rate of weight gain, internal injury and death following obstruction of the intestinal tract (Igbokwe, Rolo & Egwu 2003; Radostits *et al.* 2007). Nonmetallic indigestible foreign bodies in the reticulorumen cause recurrent rumen tympany in adult dairy cattle (Vanitha *et al.* 2010).

Ruminants reared in urban and sub-urban areas may be exposed to indigestible materials such as plastic, leather and metal. These may cause impaction, ultimately with interference of the flow of ingesta leading to rumen distension and the absence of defaecation (Abdullah, Usman & Mshelia 1984; Igbokwe *et al.* 2003; Remi-Adewunmi, Gyang & Osinowo 2004). In Jordan an estimated loss of $25 million in ruminant productivity and health associated with plastic impaction was reported (Hailat *et al.* 1996).

In Ethiopia the occurrence of foreign bodies in ruminants has been investigated and reported in the Amhara region (Sheferaw *et al.* 2014), at Jimma municipal abattoir in southwestern Ethiopia (Tesfaye, Yismaw & Demissie 2012b), in eastern Ethiopia (Tesfaye *et al.* 2012a), at Lunna export abattoir in East Shoa (Abebe & Nuru 2011), and at Addis Ababa municipal abattoir (Roman & Hiwot 2010). The objective of the present study was to estimate the prevalence and types of foreign bodies commonly encountered in cattle, sheep and goats slaughtered at Haramaya University and Haramaya municipal abattoirs in Oromia Regional State, an area of Ethiopia not previously investigated. Results were compared with those of other Ethiopian studies as well as similar studies done in Jordan (Hailat *et al.* 1996), Nigeria (Abdullah *et al.* 1984; Igbokwe *et al.* 2003; Remi-Adewunmi *et al.* 2004) and India (Vanitha *et al.* 2010).

## Research method and design

### Study area and animals

Haramaya district is located at 9°26'N and 42°3'E in Eastern Hararghe Zone, Oromia Regional State, Ethiopia. The livestock population of the study district is estimated to be 76 336 cattle, 65 083 sheep, 84 916 goats, 22 355 donkeys, 356 camels and 89 800 chickens (Central Statistical Agency 2012). The study animals included cattle, sheep and goats that were brought from Haramaya and the surrounding areas for slaughter. These animals had been kept under the traditional extensive management systems.

### Study design

A cross-sectional study was done to estimate the prevalence and types of indigestible foreign bodies ingested by ruminants in the study area. The study was conducted from November 2013 to April 2014 at Haramaya University and Haramaya municipal abattoirs.

### Sampling and sample size

The study animals were selected from cattle, sheep and goats slaughtered during each visit day using systematic random sampling. The daily cattle slaughter for both abattoirs was 20–30, whilst the daily small ruminant slaughter at Haramaya municipal abattoir was 16–28. Slaughtered cattle, sheep and goats were sampled every 2 weeks on every second day of the selected week for the study. From the daily slaughter every second animal was selected and inspected for the presence of foreign bodies. The sample size required for the study was calculated using the formula given by Thrusfield (2005). The study used 5% desired absolute precision, a 95% confidence level and 20% expected prevalence during sample size determination.

### Study methodology

Antemortem examination of selected animals was performed and the animals’ species, age, sex and body condition were recorded. Age of the animals was determined based on dental eruption as described by Gatenby (2002), Pace and Wakeman (2003) and Steele (1996). Body condition was recorded as poor, medium and good based on the appearance of the animal and manual palpation of the dorsal spines and transverse processes of the lumbar vertebrae.

After slaughter the rumen and reticulum of the selected animals were thoroughly examined externally by visual inspection and palpation. They were then carefully removed from the abdominal cavity and incised. All the contents were meticulously examined for the presence of foreign bodies which, if encountered, were examined by further dissection and the materials identified. Foreign material types were recorded for each site in each of the species.

### Data management and analysis

All collected data were entered into a Microsoft Excel spreadsheet, summarised using descriptive statistics like percentages, and the Chi square test was employed to calculate the effects of the considered risk factors (rumen versus reticulum, species and body condition) on the occurrence of foreign bodies. For analysis of the risk factors considered Stata 11.0 software (Stata 2007, Statacorp, Texas, United States of America) was used.

## Results

### Prevalence of foreign bodies

From a total of 810 ruminants (332 cattle, 285 goats and 193 sheep) examined for the presence of foreign bodies, 422 (52.1%) animals had foreign bodies in their rumen and/or reticulum. The prevalence of foreign bodies in cattle and small ruminants (sheep and goats) was 43.4% and 58.2% respectively (Table 1).

**TABLE 1:** Prevalence of foreign bodies in cattle, sheep and goats.

Species	Number examined	Number of positive animals	Prevalence (%)	95% CI	χ^2^	*p*-value
Bovine	332	144	43.4	38.0–48.7	17.53	0.000
Caprine	285	169	59.3	53.6–63.02	-	-
Ovine	193	109	56.7	49.5–63.5	-	-
Total	810	422	-	-	-	-

Between Bovine and caprine: χ^2^ = 15.56, *p* = 0.000; ovine and caprine: χ^2^ = 0.38, *p*= 0.539 and bovine and ovine χ^2^ = 8.39, *p* = 0.004.

### Foreign body types and distribution in the rumen and reticulum

The types of foreign bodies observed during this study were plastic, pieces of cloth, rope, metal and stone/calcified materials. Overall plastic materials were the most common, accounting for 79.2% of foreign bodies, whilst cloth, rope and metal accounted for 29.4%, 23.7% and 1.7% respectively. The types of foreign bodies encountered and their prevalence in the various species of ruminants are presented in Table 2. Two or more types of foreign body were observed in 43.1% of cattle, 91.7% of goats and 31.2% of sheep.

**TABLE 2:** Types and prevalence of foreign bodies in the rumen and reticulum of ruminants.

Species	Number examined	Frequency and prevalence of occurrence of foreign materials									
		Plastic		Cloth		Rope		Metal		Stone and calcified material	
		n	%	n	%	n	%	n	%	n	%
Bovine	332	87	26.2	86	25.9	33	9.9	7	2.1	8	2.4
Caprine	285	155	54.4	19	6.7	31	10.9	0	0	0	0
Ovine	193	92	47.7	19	9.8	36	18.7	0	0	0	0
Total	810	334	41.2	124	15.3	100	12.3	7	0.9	8	1.0

From a total of 422 animals with foreign bodies, in 371 (105 cattle, 161 goats and 105 sheep) the foreign material was present in the rumen, in 21 (19 cattle and 2 goats) in reticulum and in 30 animals (20 cattle, 6 goats and 4 sheep) in both rumen and reticulum. The types of foreign bodies, their distribution in rumen and reticulum, and their proportions in the three species are shown in Table 3.

**TABLE 3:** Types and proportions of foreign bodies in the rumen and reticulum of ruminant species.

Organ	Type of foreign body	Ruminant species					
		Cattle (n = 105)		Goats (n = 161)		Sheep (n = 105)	
		n	%	n	%	n	%
Rumen only	Plastic	65	74.7	148	95.5	88	95.7
	Pieces of cloth	69	80.2	15	78.9	17	89.5
	Rope	28	84.8	28	90.3	34	94.4
	Metal	-	-	-	-	-	-
	Stone/calcified	-	-	-	-	-	-
	-	n = 19	-	n = 2	-	n = 0	-
Reticulum only	Plastic	4	4.6	1	0.65	-	-
	Pieces of cloth	4	4.7	1	5.3	-	-
	Rope	-	-	-	-	-	-
	Metal	6	85.7	-	-	-	-
	Stone/calcified	5	62.5	-	-	-	-
	-	n = 20	-	n = 6	-	n = 4	-
Both rumen and reticulum	Plastic	18	20.7	6	3.9	4	4.3
	Cloth	13	15.1	3	15.8	2	10.5
	Rope	5	15.2	3	9.7	2	5.6
	Metal	1	14.3	-	-	-	-
	Stone	3	37.5	-	-	-	-
Total animals positive 442/810		144/332		169/285		109/193	

Foreign bodies recovered from rumen were significantly higher than in the reticulum: Cattle (χ^2 ^= 332, *p* < 0.05), Sheep (χ^2 ^= 193, *p*< 0.05) and goats (χ^2 ^= 285.0, *p* < 0.05).

### Foreign body versus body condition

In cattle 72.9%, 13.2% and 13.8% of foreign bodies were recovered from the rumen, reticulum and from both rumen and reticulum respectively. In sheep 96.3% of foreign bodies were recovered from the rumen and 3.7% from both rumen and reticulum. In goats 95.3% of foreign bodies were found in the rumen, 1.2% in the reticulum and 3.6% in both rumen and reticulum. The prevalence of foreign bodies relating to the body condition of the ruminants is shown in Table 4.

**TABLE 4:** Prevalence of foreign bodies in relation to sex and body condition of cattle and small ruminants.

Factors	Level of factors	Animals examined	Animals with foreign bodies	Prevalence (%)	95% CI	χ^2^	*p*-value
Body condition	Poor	14	10	71.4	46.8–96.0	38.57	0.000
	Medium	668	377	56.4	52.7–60.2	-	-
	Good	128	35	27.3	19.6–35.1	-	-
Sex	Female	319	191	59.9	54.5–65.2	12.75	0.000
	Male	491	231	47.0	42.6–51.5	-	-

Between poor and medium, χ^2^ = 1.26, *p*= 0.262; poor and good χ^2 ^= 11.33, *p*= 0.001; medium and good χ^2 ^= 36.41, *p*= 0.000.

## Discussion

This study showed an overall foreign body prevalence of 52.1% (422/810) in ruminants slaughtered at Haramaya municipal and Haramaya University abattoirs. This finding is far higher than the reports of Sheferaw *et al.* (2014), Tesfaye *et al.* (2012a, 2012b) and Roman and Hiwot (2010) in other regions of Ethiopia, where the prevalence was 41.8% (167/400), 23.9% (92/384), 9.2% (53/576) and 44.6% (311/697) respectively. These differences in the prevalence of foreign bodies between various areas may be attributed to differences in animal management systems and the extent of foreign body management both in the rural and/or urban areas and in the grazing areas. The higher prevalence of foreign bodies in the current study area is probably related to the unrestricted and increased use of plastic bags and their improper disposal. Ingestion of foreign bodies is associated with a shortage of forage (Hailat *et al.* 1996) as well as increased pollution of grazing lands with indigestible materials (Tesfaye *et al.* 2012a). If owners do not provide supplementary feed during feed shortages, their animals are likely to face a negative energy balance that will force them to ingest unusual materials including plastic, cloth, rope and even metallic objects (Hailat *et al.* 1996).

According to Remi-Adewunmi *et al.* (2004) the occurrence of foreign bodies in sheep and goats is higher within and at the outskirts of urban areas. In the present study area the animal owners let their animals move freely to forage on the highly polluted road verges. The agricultural land in the area is largely occupied by perennial crops like the narcotic ‘chat’, which leaves reduced grazing land available for livestock. These factors increased the chance of foreign material ingestion.

The prevalence of foreign bodies observed in cattle (43.4%) was in approximate agreement with the report of Sheferaw *et al.* (2014), who reported 41.8% in cattle from the Amhara region of Ethiopia. The prevalence of foreign bodies was significantly (χ^2 ^= 17.53, *p* < 0.05) higher in sheep (59.3%) and goats (56.7%) than in cattle (43.4%), but there was no significant difference between sheep and goats. This finding was inconsistent with those of Sheferaw *et al.* (2014), who reported 41.8% (167/400) in cattle, 20.6% (66/320) in sheep and 11.9% (38/320) in goats. The absence of significant differences in the prevalence of foreign bodies in sheep and goats is in agreement with the report of Abebe and Nuru (2011), who reported 7.55% (29/384) in sheep and 4.68% (18/384) in goats.

Plastic was the most commonly encountered (79.2%) foreign material in all study animals, followed by cloth (15.3%) and rope (12.3%). This finding is in general agreement with various reports from different areas of Ethiopia (Abebe & Nuru 2011; Roman & Hiwot 2010; Sheferaw *et al.* 2014; Tesfaye *et al.* 2012b), Nigeria (Igbokwe *et al.* 2003; Remi-Adewunmi *et al.* 2004) and Jordan (Hailat *et al.* 1996). This indicates the widespread use of plastic bags in these areas and environmental pollution due to their improper disposal.

In all of the ruminants more foreign bodies occurred in the rumen alone (87.9%) than in the reticulum alone (5.0%). Significantly higher proportions of foreign bodies were observed in the rumen of cattle (χ^2 ^= 332, *p*< 0.05), sheep (χ^2 ^= 193, *p*< 0.05) and goats (χ^2 ^= 285.0, *p*= 0.000) than in the reticulum. This finding was in general agreement with the findings of Abebe and Nuru (2011), Roman and Hiwot (2010) and Tesfaye *et al.* (2012b), and may be attributed to the larger rumen volume, the cumulative size/s and material composition of the foreign bodies, and the types of materials, with metals and sharp objects tending to localise preferentially in reticulum (Radostits *et al.* 2007).

Overall the prevalence of foreign bodies in study animals with poor body condition of 71.4% (10/14) was significantly higher (χ^2 ^= 38.57, *p* < 0.05) than in those with medium (56.4% or 377/668) and good body condition (27.3% or 35/128). This finding is in agreement with the reports of Abebe and Nuru (2011) and Hailat *et al.* (1996). However, the total number of study animals in poor condition was small at 1.7% (14/810). The majority of animals with foreign bodies were in the medium-condition group (82.4% or 668/810 of the animals examined, with 56.4% [377/668] having foreign bodies), whilst 15.8% (128/810) of animals were in good body condition and 27.3% (35/128) of these were also positive.

Accumulations of indigestible foreign bodies in the rumen interfere with the flow of ingesta (Igbokwe *et al.* 2003) and with absorption of feed. These effects most likely contributed significantly to animals being in poor body condition, and may have played a significant role in preventing a percentage of those in medium body condition who had foreign bodies from attaining good body condition, and thus causing their owners to incur significant financial losses.

## Conclusion and recommendations

The prevalence of foreign bodies observed in the rumen and reticulum of Ethiopian cattle, sheep and goats presenting at the Haramaya University and Haramaya municipal abattoirs was high at 52.1%. The study showed that improper disposal of and pollution of the environment with indigestible materials like plastic, cloth, metal, rope and stone could pose serious health risks for free-grazing ruminants. Amongst the foreign bodies observed, plastic bags constituted the majority. Based on the results obtained, the use of biodegradable paper bags could be encouraged whilst the community is educated in the principals of re-use, recycling and responsible waste disposal that will not put the environment and animals at risk.

Livestock professionals and environmental activists are the key actors in educating the community on the risk of improper disposal of plastic and other indigestible materials in an ecosystem where free communal grazing of animals is practised. Policy makers are also expected to formulate a legally binding regulatory framework towards reducing environmental pollution and also on the extent to which the free-grazing system is practised.
